# The Role of Toll-Like Receptors in Hematopoietic Malignancies

**DOI:** 10.3389/fimmu.2016.00390

**Published:** 2016-09-28

**Authors:** Darlene A. Monlish, Sima T. Bhatt, Laura G. Schuettpelz

**Affiliations:** ^1^Department of Pediatrics, Washington University School of Medicine, St. Louis, MO, USA

**Keywords:** toll-like receptors, MyD88, HSC, myelodysplastic syndrome, leukemia

## Abstract

Toll-like receptors (TLRs) are a family of pattern recognition receptors that shape the innate immune system by identifying pathogen-associated molecular patterns and host-derived damage-associated molecular patterns. TLRs are widely expressed on both immune cells and non-immune cells, including hematopoietic stem and progenitor cells, effector immune cell populations, and endothelial cells. In addition to their well-known role in the innate immune response to acute infection or injury, accumulating evidence supports a role for TLRs in the development of hematopoietic and other malignancies. Several hematopoietic disorders, including lymphoproliferative disorders and myelodysplastic syndromes, which possess a high risk of transformation to leukemia, have been linked to aberrant TLR signaling. Furthermore, activation of TLRs leads to the induction of a number of proinflammatory cytokines and chemokines, which can promote tumorigenesis by driving cell proliferation and migration and providing a favorable microenvironment for tumor cells. Beyond hematopoietic malignancies, the upregulation of a number of TLRs has been linked to promoting tumor cell survival, proliferation, and metastasis in a variety of cancers, including those of the colon, breast, and lung. This review focuses on the contribution of TLRs to hematopoietic malignancies, highlighting the known direct and indirect effects of TLR signaling on tumor cells and their microenvironment. In addition, the utility of TLR agonists and antagonists as potential therapeutics in the treatment of hematopoietic malignancies is discussed.

## Introduction

Toll-like receptors (TLRs) are a family of pattern recognition receptors (PRRs) that play a central role in innate immunity. TLRs are expressed on a wide variety of effector immune and stromal cell types, as well as hematopoietic stem and progenitor cells (HSPCs) (1–3), and signaling through these receptors is essential to mounting an effective immune response to an acute infection or injury. However, while TLR signaling is important to the normal immune response, enhanced or aberrant TLR signaling is associated with ineffective hematopoiesis and hematopoietic malignancy (4, 5). Most notably, enhanced expression of components of the TLR signaling pathway, as well as activating mutations in some cases, are associated with lymphoproliferative disorders and myelodysplastic syndromes (MDS) (6–16).

This review focuses on the contribution of TLR signaling to impaired hematopoiesis and hematopoietic malignancy. We will explore the role of cell-autonomous aberrant TLR signaling to loss of normal HSPC function and leukemogenesis, as well as the non-cell-autonomous effects of TLR signaling that may contribute to disease pathogenesis. Finally, we will discuss the utility of TLRs and their downstream effectors as therapeutic targets in hematopoietic neoplasms.

## TLR Signaling Overview

A total of 12 TLR family members have been described in mice, and 10 in humans (17). In addition to identifying foreign pathogen-associated molecular patterns [PAMPS, e.g., peptidoglycans, lipopolysaccharide (LPS)], TLRs recognize a wide variety of non-pathogen-associated ligands. These so-called damage-associated molecular patterns (DAMPS) include various endogenous by-products of cellular damage, such as free nucleic acids and extracellular matrix components (18). TLRs are widely expressed on both immune cells and non-immune cells, including dendritic cells, macrophages, lymphocytes, HSPCs, and endothelial cells (3, 19–22). Some of the TLRs are localized to the plasma membrane (TLR1, TLR2, TLR4, TLR5, TLR6, and TLR11), while others are found in endosomes (TLR3, TLR7, TLR8, and TLR9). In general, the TLRs act as homodimers, with the exception of TLR2, which heterodimerizes with TLR1 or TLR6 (23–25). Each TLR has a unique profile of known ligands, dictated in part by their subcellular localization.

Signaling through TLRs requires the recruitment of intracellular adaptor proteins, with all TLRs, except TLR3, using the myeloid differentiation primary response gene 88 (MyD88). Following recruitment of MyD88 to activated TLRs, this adaptor complexes with the serine–threonine kinase interleukin-1 receptor-associated kinase 4 (IRAK4) (26), which in turn recruits other IRAK family members (IRAK1 and IRAK2), forming a complex referred to as the “Myddosome” (27). IRAK1 interacts with the tumor necrosis factor (TNF) R-associated factor 6 (TRAF6) E3 ubiquitin ligase. TRAF6 then participates in the activation of transforming growth factor beta-activated kinase 1 (TAK1), which in turn activates the nuclear factor k-light-chain-enhancer of activated B cells (NF-κB) and mitogen-activated protein kinase (MAPK) pathways and promotes the expression of various proinflammatory cytokines (e.g., interleukin-1 (IL-1), IL-6, IL-8, and TNFα) (28).

**Figure 1:**
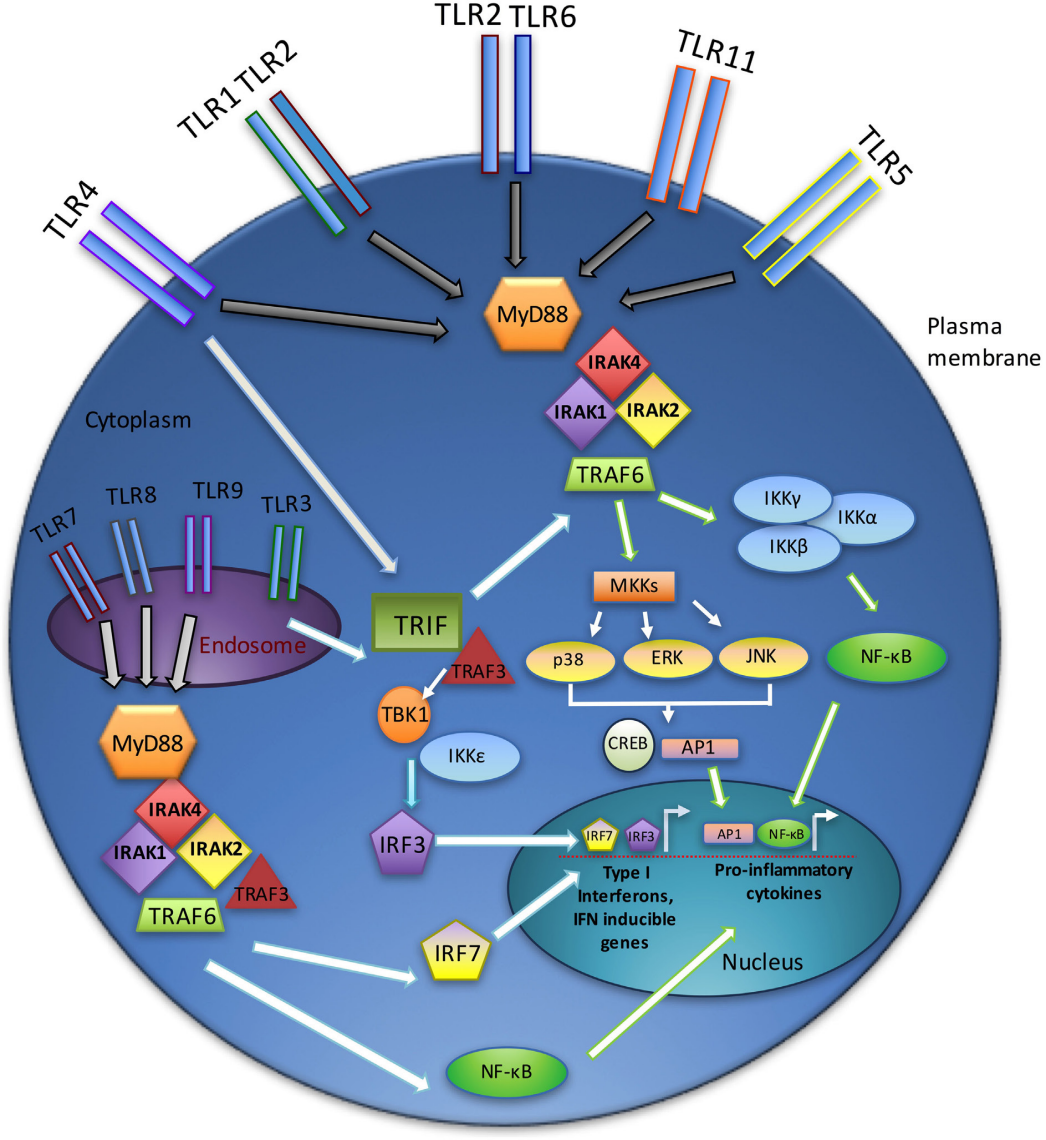
**TLR signaling overview**. TLR signaling may be divided into the MyD88-dependent and MyD88-independent (TRIF-dependent) signaling pathways. In the MyD88-dependent pathway, activation of MyD88 from membrane-bound TLR ligation forms complexes with IRAKs1, 2, and 4, leading to an interaction with TRAF6. Ultimately, the NF-κB and MAPK pathways are activated, resulting in the production of inflammatory cytokines. The TRIF-dependent (MyD88-independent) pathway requires TRIF binding to TRAF3, which recruits TBK1 and IKKε, thereby activating IRF3. Endosomal TLRs (3, 7, 8, and 9) act through the IRAK–TRAF6 complex to activate IRF7. Together, IRF3 and IRF7 stimulate type I interferon production.

Toll-like receptor3 functions independently of MyD88, utilizing instead the adaptor protein TIR-domain-containing adaptor-inducing interferon-B (TRIF) (29). TRIF, upon binding to TRAF3, recruits the IKK-related kinases TBK1 and IKKε and ultimately activates interferon regulatory factor-3 (IRF3) and stimulates the production of type I interferons (IFNs) (30). In addition, TRIF interacts with TRAF6 and contributes to MyD88-independent activation of NF-κB and MAPKs (31). TLR4 uniquely utilizes both MyD88- and TRIF-dependent pathways (Figure 1) (32).

## TLR Signaling and Hematopoietic Stem and Progenitor Cells

In response to acute infection or injury, TLR signaling promotes the production of proinflammatory cytokines and assists with the development of the adaptive immune response. Recent studies suggest that outside of their well-known role in responding to pathogens and other insults by stimulating effector immune cells, TLRs can influence hematopoiesis from the level of the hematopoietic stem cell (HSC). Both mouse and human HSCs express TLRs (1–3), and accumulating data demonstrate that TLR ligands can influence the proliferation, mobilization, and differentiation of HSCs and committed progenitors (2, 4, 5, 33–37). In fact, recent studies have shown that proinflammatory signals, including TLR4–MyD88–NF-κB stimulation, are necessary for HSPC emergence in zebra fish and mouse embryos (38, 39). Thus, TLR signaling contributes to early hematopoiesis and continues to influence HSPCs into adulthood. Notably, sustained exposure to TLR signals is associated with loss of normal HSC function. Chronic *in vivo* treatment of mice with the TLR4 agonist LPS, for example, leads to HSC cycling and a promotion of myeloid differentiation with a loss of HSC repopulating activity in transplantation experiments (4). Similarly, systemic exposure of mice to the TLR2 agonist PAM_3_CSK_4_ leads to an expansion of bone marrow and spleen phenotypic HSCs, but a loss of bone marrow HSC self-renewal (40). Conversely, loss of TLR signaling (in *Tlr2^−/−^, Tlr4^−/−^, Tlr9^−/−^*, or *MyD88^−/−^* deficient mice) is associated with enhanced HSC repopulating activity (41, 42). This last point illustrates that even in the absence of overt infection or other insult, TLR signaling regulates baseline hematopoiesis.

The effects of TLR signaling on HSPCs are likely mediated by both cell-autonomous and cell non-autonomous mechanisms. Evidence for a direct, cell-autonomous role for TLR signaling in regulating HSCs comes from *in vitro* studies showing that incubation of HSPCs with TLR agonists induces cell cycling and myeloid differentiation (1, 2). Furthermore, Megias and colleagues demonstrated that purified HSPCs (c-Kit+ Sca-1+ Lineage− IL7Ra− cells) from wild-type mice transplanted into *Tlr2^−/−^, Tlr4^−/−^*, or *MyD88^−/−^* recipients differentiated into macrophages in response to specific TLR ligands (37). This approach, by removing a potential contribution of soluble mediators of the effects of TLR signals from other hematopoietic or stromal cells, supports the idea that TLR ligands can have direct, cell-autonomous effects on HSPCs *in vivo*. In agreement with this idea, we recently reported that systemic exposure of mice to a TLR2 ligand regulates HSCs, in part, *via* cell-autonomous TLR2 signaling (40). Chimeric mice were generated in which a mixture of *Tlr2^−/−^* and WT bone marrow was transplanted into lethally irradiated *Tlr2^−/−^* recipients. Treatment of these chimeras with the TLR2 agonist PAM_3_CSK_4_ led to a relatively greater expansion of WT HSCs compared to *Tlr2^−/−^* HSCs, supporting a role for cell-autonomous TLR2 signaling in regulating HSCs.

While *in vitro* and chimeric animal studies endorse a role for cell-autonomous TLR signaling in the regulation of immature hematopoietic cells, a large number of proinflammatory cytokines are produced by effector immune cells and progenitors in response to TLR ligands, and these cytokines are known to influence HSPC cycling, differentiation, survival, and function, as well (43). For example, several recent studies have shown that systemic TLR4 stimulation in mice promotes HSC mobilization indirectly *via* production of granulocyte-colony stimulating factor (G-CSF) by endothelial cells (36, 44). Systemic TLR2 stimulation in mice also leads to G-CSF production, as well as increased serum levels of TNFα, and inhibition of these cytokines partially rescues the proliferation and mobilization of HSPCs in response to TLR2 agonist treatment (40). Finally, using a microfluidic single-cell proteomics platform, Zhao et al. showed that TLR2 and TLR4 ligands stimulate abundant cytokine production by short-term HSCs and multipotent progenitors, which in turn promote myeloid differentiation in an autocrine or paracrine manner (45). Thus, TLR ligands influence HSPC cycling, differentiation, and mobilization both directly, *via* cell-autonomous signaling, and indirectly, *via* non-cell-autonomous production of proinflammatory cytokines.

## TLR Signaling and Hematopoietic Malignancy

As noted above, sustained or dysregulated TLR signaling, *via* direct cell-autonomous as well as indirect mechanisms involving inflammatory cytokines, may contribute to loss of normal HSC function. Furthermore, numerous recent studies have demonstrated enhanced TLR expression and signaling in hematopoietic malignancies (Table 1). For example, activated TLR signaling and overexpression of TLRs and their downstream effectors are associated with MDS (11–15), a heterogeneous group of HSC disorders associated with ineffective hematopoiesis, myeloid dysplasia, and a high risk of transformation to acute leukemia. In addition, an activating mutation in MyD88 is commonly found in lymphoid malignancies (6–10, 46). While the exact role for this enhanced TLR signaling in these malignancies is not clear, it likely has, as with normal HSPCs, both cell-autonomous and non-autonomous effects on premalignant and malignant cells. Below, we discuss, in further detail, our current understanding of the role of TLR signaling in these and other hematopoietic malignancies and discuss the potential utility of TLR signaling as a therapeutic target in their treatment.

**Table 1:** **Overview of toll-like receptors associated with hematologic malignancies**.

Toll-like receptor	Hematologic malignancy	Reference
TLR1	Myelodysplastic syndrome (MDS), acute lymphoblastic leukemia (ALL), acute myeloid leukemia (AML), multiple myeloma (MM), chronic lymphocytic leukemia (CLL)	MDS: (15, 53); ALL: (54); AML: (63, 64); MM: (72, 73); CLL: (75)
TLR2	Myelodysplastic syndrome (MDS), acute lymphoblastic leukemia (ALL), acute myeloid leukemia (AML), multiple myeloma (MM), chronic lymphocytic leukemia (CLL)	MDS: (15, 53, 57, 59, 60); ALL: (54); AML: (62, 64); MM: (71, 73); CLL: (75)
TLR3	Acute myeloid leukemia (AML), multiple myeloma (MM), indolent non-Hodgkin’s lymphoma (iNHL)	AML: (64, 88); MM: (72, 73, 87); iNHL: (104)
TLR4	Myelodysplastic syndrome (MDS), acute myeloid leukemia (AML), multiple myeloma (MM), Mantle cell lymphoma (MCL), Follicular non-Hodgkin’s lymphoma (NHL)	(11, 52, 57, 59–61); AML: (62, 64); MM: (71–73); MCL: (74); NHL: (103)
TLR5	Acute myeloid leukemia (AML), multiple myeloma (MM)	AML: (64); MM: (73)
TLR6	Myelodysplastic syndrome (MDS), acute lymphoblastic leukemia (ALL), acute myeloid leukemia (AML), multiple myeloma (MM), chronic lymphocytic leukemia (CLL)	MDS: (15, 53); ALL: (54); AML: (64); MM: (73); CLL: (75)
TLR7	Acute myeloid leukemia (AML), activated B-cell (ABC) diffuse large B-cell lymphoma (DLBCL), multiple myeloma (MM), chronic lymphocytic leukemia (CLL)	AML: (64, 86); ABC DLBCL: (70); MM: (72, 73); CLL: (75)
TLR8	Acute myeloid leukemia (AML), multiple myeloma (MM)	AML: (64, 86); MM: (72, 73)
TLR9	Acute myeloid leukemia (AML), activated B-cell (ABC) diffuse large B-cell lymphoma (DLBCL), multiple myeloma (MM), chronic lymphocytic leukemia (CLL), B-cell lymphoma (BCL)	AML: (64); ABC DLBCL: (70); MM: (71–73); CLL: (75); BCL: (102)
TLR10	Acute myeloid leukemia (AML), multiple myeloma (MM), chronic lymphocytic leukemia (CLL)	AML: (64); MM: (73); CLL: (75)
TLR11	Acute myeloid leukemia (AML), multiple myeloma (MM)	AML: (64); MM: (73)
TLR12	Acute myeloid leukemia (AML), multiple myeloma (MM)	AML: (64); MM: (73)
TLR13	Acute myeloid leukemia (AML), multiple myeloma (MM)	AML: (64); MM: (73)

## Aberrant TLR Signaling in MDS

Myelodysplastic syndromes are a heterogeneous group of clonal HSC disorders characterized by peripheral cytopenias, myeloid dysplasia, and a high risk of transformation to acute leukemia (47). MDS is one of the most common hematopoietic malignancies of older adulthood, and it occurs less frequently in children and young adults. The disease may occur *de novo*, or may arise following prior cytotoxic therapies. Distinct subtypes of MDS are identified based on genetic and morphological features, and in children MDS is often associated with inherited bone marrow failure syndromes (48). Regardless of the underlying genetic landscape, deregulation of innate immune signaling, and, in particular, enhanced TLR signaling, is associated with MDS (49, 50). Thus, TLRs and their downstream effectors are attractive therapeutic targets in MDS, as stem cell transplantation is presently the only curative therapy and is only feasible for a subset of patients (51).

Increased expression of multiple TLRs and signaling intermediates has been reported in MDS, as well as a loss of TLR signaling repressors. In patients with the deletion 5q (del (5q)) subtype of MDS, for example, loss of two micro-RNAs, miR-145 and miR-146a, which normally inhibit the TLR signaling intermediates TIR-domain-containing adaptor protein (TIRAP that associates with MyD88 and assists in the formation of the Myddosome) and TRAF6, leads to inappropriate activation of TLR signaling (14). This, in turn, results in excessive IL-6 production, which contributes to ineffective hematopoiesis, including thrombocytosis, megakaryocyte dysplasia, and neutropenia. Additionally, enforced expression of TRAF6 or knockdown of miR-145 or miR-146 in murine HSCs phenocopies some of the features of del (5q) MDS, suggesting that inappropriately activated TLR pathway signaling within the HSCs contributes to disease pathogenesis (14). Recently, Varney et al. (13) described an additional mechanism for increased TRAF6 expression in del (5q) MDS involving haploinsufficiency for TRAF-interacting protein with forkhead-associated domain B (TIFAB). TIFAB forms a complex with TRAF6, reducing its stability by a lysosome-dependent mechanism, and mice transplanted with *Tifab^−/−^* HSPCs display a bone marrow failure like disease with cytopenias and myeloid differentiation skewing. Furthermore, re-expression of TIFAB in human MDS/AML cell lines with low endogenous TIFAB significantly reduced leukemic colony formation and cell survival. Thus, along with miR-146a, TIFAB helps to limit TRAF6 expression and regulate HSPC differentiation and function.

In addition to the data supporting a role for enhanced TLR signaling in del (5q) MDS, numerous studies have shown increased expression of TLRs and TLR pathway intermediates in patients with all types of MDS. For example, Maratheftis and colleagues showed increased TLR4 expression in the CD34+ cells of patients with MDS in a small series of 21 cases, and this expression was associated with increased apoptosis (11). Velegraki and colleagues (52) found increased expression of TLR4 and upregulation of TLR4-mediated signaling in the CD14+ bone marrow cells of patient with MDS. Furthermore, they reported that impaired clearance of apoptotic cells by macrophages in the bone marrow of patients with MDS is associated with increased circulating levels of the endogenous TLR agonist high mobility group protein B1 (HMGB1), and they concluded that this contributes to enhanced proinflammatory cytokine production by monocytes and impaired clonogenic potential of CD34+ cells in MDS patients. More recently, Wei and colleagues demonstrated increased expression of TLR2 and its functional binding partners, TLR1 and TLR6, in the CD34+ cells of a series of 149 patients with MDS (15). Furthermore, they identified an acquired, activating variant of TLR2 (TLR2-F217S) in 11% of their cohort of individuals with MDS, which is associated with enhanced NF-κB activation in response to TLR2 ligand exposure. This group showed that inhibition of TLR2 in primary cultured bone marrow CD34+ cells from patients with MDS by short hairpin RNA led to improved erythroid colony formation *in vitro*, further supporting a role for this receptor in the pathogenesis of MDS. They proposed a model whereby augmented TLR2 signaling leads to activation of the histone demethylase JMJD3, thus stimulating the abnormal epigenetic activation of multiple innate immunity genes, including IL-8, and promoting ineffective hematopoiesis. Based on these data, a phase I/II study of an anti-TLR2 antibody (OPN-305) is underway for second-line treatment of lower-risk MDS patients (Opsona Therapeutics, Dublin, Republic of Ireland).

Interestingly, while expression of TLR2 itself in the above study correlated with low-risk disease by the International Prognostic Scoring System and longer survival, higher expression of its binding partner, TLR6, was associated conversely with higher-risk disease and increased bone marrow blasts (15). This finding raises the intriguing possibility that the effects of TLR2 signaling in MDS may be influenced by which heterodimer pair is stimulated (TLR1/2 versus TLR2/6). Historically, this association with different partners has been thought to expand the ligand spectrum without altering downstream signaling (53). However, more recent data suggest that there are heterodimer-specific differences with respect to activation of downstream pathways. For example, Rolf and colleagues (54) demonstrated that stimulation of acute lymphoblastic leukemia (ALL) cell lines and primary ALL samples with the specific TLR1/2 agonist, PAM_3_CSK_4_, versus the TLR2/6 agonist, PAM_2_CSK_4_, led to distinct activation kinetics of the NF-κB and PI3K pathways. Furthermore, only PAM_3_CSK_4_ sensitized ALL cells to vincristine-mediated toxicity. Further studies are needed to determine whether there are heterodimer-specific effects of TLR2 stimulation on MDS cells, as this could have implications for the development of targeted therapies.

In addition to the TLRs themselves, the expression of numerous downstream signaling mediators is increased in patients with MDS, as well. For example, increased expression of the TLR signaling adaptor, MyD88, was found in 40% of individuals in a cohort of 64 patients with MDS, and high expression correlated with shorter survival (16). Similarly, IRAK1 is overexpressed in the CD34+ cells of approximately 10–30% of patients with MDS, with high expression associated with reduced overall survival (12, 55, 56). Pharmacologic or genetic inhibition of IRAK1 induced cell cycle arrest and apoptosis in MDS cells and improved survival in an MDS xenograft model (56), suggesting that IRAK1 may be a potential therapeutic target in MDS.

Thus, multiple studies have linked increased TLR signaling to MDS; however, the specific contribution of this signaling to disease progression is not clear. The existing data suggest that aberrant TLR signaling promotes ineffective hematopoiesis and likely contributes to the cytopenias characteristic of the disease. However, it is not at all clear whether this aberrant signaling promotes the transformation of MDS to acute leukemia. Further studies will be necessary to determine if TLR expression and signaling in premalignant MDS HSCs changes upon transformation, and whether TLR stimulation contributes to the acquisition of clonal dominance in this disease. In addition, it is not clear to what extent enhanced TLR signaling outside of the CD34+ population contributes to MDS pathogenesis. Accumulating evidence supports a role for the bone marrow microenvironment in the evolution of MDS, with numerous alterations in the bone marrow stromal and hematopoietic populations and deregulation of inflammatory cytokine secretion contributing to ineffective hematopoiesis. For example, myeloid-derived suppressor cells (MDSCs), an immature myeloid population with potent T-cell suppressive capabilities, are expanded in the bone marrow of MDS patients (57, 58). MDSCs were first characterized in tumor-bearing mice and patients with cancer and contribute to immune escape of malignant cells. Notably, S100A9, which is a DAMP that is increased in the plasma of MDS patients and binds TLR2 and TLR4, has been shown to stimulate the expansion and activation of MDSCs (57, 59, 60). S100A9 transgenic mice display an accumulation and activation of MDSCs and develop progressive multilineage cytopenias and dysplastic features characteristic of human MDS (57). Schneider and colleagues (61) also demonstrated a link between haploinsufficiency of the ribosomal gene, *Rps14*, which has been linked to the severe macrocytic anemia observed in del (5q) MDS patients, to activation of the calcium binding proteins, S100A8 and S100A9. The authors found that S100A8, acting upstream of TLR4 and TNFα, contributed to a p53-dependent erythroid-differentiation defect. Further studies are needed to comprehensively define the different bone marrow populations in which aberrant TLR signaling occurs in MDS and to determine the relative contribution of cell-autonomous versus cell non-autonomous signaling to the disease pathogenesis.

## TLR Signaling in Other Myeloid Malignancies

In addition to MDS, aberrant TLR expression has been associated with other myeloid neoplasms. For example, in a study of 103 newly diagnosed patients with acute myeloid leukemia (AML), Rybka and colleagues found that the mRNA expression of TLR2 and TLR4 was significantly higher in the bone marrow of patients with myelomonocytic and monoblastic acute leukemia compared to those with other types of AML. Furthermore, the expression of these receptors was also higher in patients with no response to induction therapy compared to those who achieved complete remission, and the overall survival of patients with higher TLR2 and TLR4 expression was significantly shorter than that of patients with lower expression (62). One of the binding partners of TLR2, TLR1, was also shown to be significantly upregulated in leukemic stem cells (LSCs) derived from 18 AML patients as compared to normal bone marrow cells derived from healthy donors, a finding consistent with elevated TLR1 mRNA levels observed in MDS patients described earlier (15, 63). The authors stimulated LSCs both *in vitro* and *ex vivo* with the TLR2/1 agonist, PAM_3_CSK_4_, and found that agonist treatment led to expansion, increased leukemic cell survival, and a shift toward differentiation. Similarly, in a study of mRNA expression of TLRs in myeloid leukemia cell lines, Okamoto and colleagues found that 10 TLRs were expressed in the majority of leukemic cell lines assessed (64). The authors evaluated the functionality of the TLRs by examining cell proliferation and differentiation upon ligand exposure. Ligand stimulation, however, failed to trigger differentiation (contrary to what was observed in normal bone marrow progenitors), thereby warranting further study of the mechanism through which TLR signaling induces differentiation in normal and malignant cells.

## TLR Signaling in Lymphoid Malignancies

In addition to myeloid malignancies, enhanced TLR signaling has been implicated in a number of lymphoid neoplasms as well. Notably, lymphoid malignancies are commonly associated with a specific gain-of-function mutation in MyD88. This single nucleotide variant leads to the substitution within the MyD88 TIR domain of a leucine residue at position 265 to a proline (L265P), which ultimately promotes enhanced IRAK1 phosphorylation and activation of NF-κB and JAK–STAT3 signaling with production of proinflammatory cytokines and promotion of cell survival (6, 65). MyD88 (L265P) is highly recurrent, with 29% of a cohort of 382 patients with activated B-cell-like diffuse large B-cell lymphoma possessing this mutation (6). The same mutation was found in 9% of gastric mucosa-associated lymphoid tissue (MALT) lymphomas. It was also identified in 2.9% of patients with chronic lymphocytic leukemia and was associated with younger age and more advanced clinical stage (7). Finally, the L265P MyD88 mutation was found in nearly 50% of patients with immunoglobulin M (IgM) monoclonal gammopathy of unknown significance (MGUS) and >90% (66) of patients with Waldenstrom macroglobulinemia (WM) (9, 10). In addition to activating IRAK activity, Yang and colleagues (67) found that the MyD88 (L265P) variant supports lymphoplasmacytic cell survival in WM through stimulation of Bruton tyrosine kinase (BTK), and suppression of BTK and IRAK1/2 had synergistic effects on inhibiting NF-κB signaling and promoting apoptosis in WM cells. In a study utilizing a retroviral gene transfer system in mice, Wang and colleagues (68) demonstrated that the MyD88 (L265P) variant alone was capable of driving B cell division *in vitro* in the absence of exogenous TLR ligands. However, this proliferation was dependent upon intact signaling by the RNA/DNA-sensing TLRs, as it was inhibited by the *Unc93b1^3d^* mutation (which disrupts exogenous antigen presentation and signaling *via* TLRs 3, 7, and 9) (66, 69), TLR9 deficiency, and chloroquine exposure (necessary for proteolytic activation of TLR ectodomains). The dependence of oncogenic MyD88 on TLRs has also been suggested in activated B-cell (ABC) diffuse large B-cell lymphoma (DLBCL), where both the depletion of a number of proteins necessary for TLR7 and TLR9 trafficking and signaling, including UNC93B1, CD14, and PRAT4A, and pharmacologic inhibitors of these TLRs led to cell death *in vitro* (70).

In addition to the activating MyD88 (L265P) mutation, the expression of TLR signaling pathway components is increased in lymphoid malignancies. For example, TLRs have been found to be more highly expressed in the plasma cells obtained from multiple myeloma patients as compared to healthy control donors (71). Additionally, culture of human myeloma cell lines and primary cells with TLR ligands stimulates cell growth and spares these cells from serum deprivation or dexamethasone-induced apoptosis *via* autocrine secretion of interleukin-6 (IL-6) (71–73). In a study of Mantle cell lymphoma (MCL), an incurable B-cell malignancy, Wang and colleagues found that the levels of TLR4 expression were significantly higher on primary MCL cells than normal peripheral blood mononuclear cells (PBMCs) or B cells, and activation of TLR4 by LPS promotes tumor growth and enables MCL cells to evade the immune system *via* secretion of IL-6, IL-10, and vascular endothelial growth factor (VEGF) (74). A study of TLR expression patterns in purified leukemic B-lymphocytes isolated from chronic lymphocytic leukemia (CLL) patients and the MEC1 CLL cell line revealed an increased expression of TLRs 1, 2, 6, 7, 9, and 10 (75). Further *in vitro* studies demonstrated that the TLR2/1 and TLR2/6 ligands, PAM_3_CSK_4_ and MALP-2 (mycoplasmal macrophage-activating lipopeptide-2), protected CLL cells from spontaneous apoptosis. Finally, single nucleotide polymorphisms (SNPs) in several TLRs have been associated with lymphoma risk (76–79).

## TLR Signaling in Non-Hematopoietic Malignancies

While a detailed discussion of the role of TLR signaling in non-hematopoietic malignancies is outside the scope of this review, cell-intrinsic signaling for a number of TLRs has been associated with promoting oncogenesis (80). For example, TLR2 has recently been shown to have a cell-intrinsic role in regulating epithelial cell growth and oncogenesis. Scheeren and colleagues (81) reported that loss of TLR2 signaling in intestinal epithelium reduces damage-induced colitis regeneration and spontaneous tumor formation in *Apc*^min/+^ mice [carrying a point mutation in the adenomatous polyposis coli (Apc) gene, which leads to development of intestinal tract tumors]. Furthermore, TLR2 is expressed on breast epithelia, and TLR2 agonist treatment of luminal and basal breast epithelial cells promotes *in vitro* colony growth. Conversely, TLR2 loss inhibited the growth of breast cancer cells both *in vitro* and *in vivo*. Another recent study showed that STAT3-mediated upregulation of TLR2 expression on gastric epithelial cells promoted cell proliferation and survival, and inhibition of TLR2 suppressed gastric tumor formation (82). Cherfils-Vicini et al. (83) demonstrated expression of TLR7 and TLR8 in human primary lung cancer sections and lung tumor cell lines and showed that stimulation of these cell lines in culture with TLR7 and TLR8 agonists led to activation of NF-κB, as well as increased expression of the anti-apoptotic protein Bcl-2, enhanced survival, and chemoresistance. Additionally, He and colleagues described the expression of functional TLR4 in human lung cancer cells (84) and found that TLR4 stimulation led to the secretion of numerous immunosuppressive cytokines, including VEGF and TGF-β, and the proangiogenic chemokine IL-8, thereby inducing resistance to TNFα and TRAIL-induced apoptosis *in vitro*. Finally, high TLR9 expression was also observed in both primary lung cancer specimens and tumor cell lines (85), and activation of the TLR9 pathway resulted in the production of monocyte chemoattractant protein-1 (MCP-1) and the reduction of TNFα-induced apoptosis. Together, these data from non-hematopoietic malignancies support a role for cell-autonomous TLR signaling in promoting the growth and survival of malignant cells.

## TLRs as Therapeutic Targets in Hematopoietic Malignancy

Treatment of hematopoietic malignancies is still largely based on chemotherapeutic drug regimens and stem cell transplantation, and new therapies are needed. The association of enhanced TLR expression and signaling with hematopoietic malignancies suggests that inhibition of these receptors or their downstream effectors may confer therapeutic benefit. As discussed above, for example, a phase I/II study of an anti-TLR2 antibody (OPN-305, Opsona Therapeutics, Dublin, Ireland) is currently underway for the second-line treatment of lower-risk MDS patients based on preclinical studies showing improved erythroid colony formation upon TLR2 inhibition in MDS CD34+ cells *in vitro*. Similarly, as the activating MyD88 L265P mutation is prevalent in multiple lymphoid malignancies, particularly WM, inhibiting this factor represents an attractive therapeutic strategy. Indeed, preclinical studies have shown that loss of MyD88 or its downstream targets (BTK, IRAK1, IRAK4) suppresses NF-κB signaling and induces WM cell death. Ibrutinib is a BTK inhibitor that was recently approved for the treatment of WM following a prospective, multicenter trial that showed its efficacy with high response rates and improved progression-free survival in previously treated patients (46, 86).

Although the role of TLR signaling in hematopoietic malignancies is largely thought to be that of promoting disease, as reviewed above, and inhibitors of TLR signaling, such as OPN-305 and Ibrutinib, currently offer therapeutic promise, accumulating evidence actually supports a potential role for TLR agonists in the treatment of some hematopoietic malignancies as well. Recent work by Ignatz-Hoover and colleagues (87), for example, demonstrated that the TLR7/8 agonist R848 promotes *in vitro* terminal differentiation and inhibits proliferation of AML cell lines and primary patient samples in a TLR8–MyD88–p38-dependent fashion. Furthermore, R848 significantly inhibited AML tumor growth in an AML NSG mouse xenograft model. In human myeloma cell lines, stimulation with polyinosinic–polycytidylic acid (poly I:C), an agonist of TLR3, led to increased apoptosis and induced activation of the NFκB pathway and type I IFN secretion, thereby inhibiting cell growth. Poly I:C also led to TLR3 upregulation, type I IFN induction, and increased apoptosis in primary AML cells (88, 89). Similarly, treatment of ALL cell lines and primarily ALL samples with the specific TLR2/1 ligand, PAM_3_CSK_4_, triggered caspase-8-mediated apoptosis and sensitized ALL cells to vincristine-mediated toxicity *in vitro* (54). These studies support the idea that cell-autonomous TLR signaling may, in fact, have an antitumor effect in some hematopoietic malignancies.

In addition to a potential direct antitumor effect, TLR agonists are being used in multiple tumor types, including hematopoietic malignancies, for their ability to elicit anticancer immune responses (90–95). In this respect, TLR agonists act to disrupt tolerance to tumor cells, thus facilitating the destruction of these cells by the patient’s own immune system. As early as the 1960s, Mathe and colleagues described enhanced survival in pediatric leukemia patients treated with injections of the TLR agonist, Bacille Calmette–Guérin (BCG) bacilli, which is still currently utilized for the treatment of bladder cancer (96–98). The majority of clinical trials utilizing TLR agonists have focused on TLR3, TLR7/8, and TLR9, and recent studies have indicated that the combinatorial use of TLR agonists may be beneficial for patients whose tumors express multiple TLRs (99–101). With respect to hematologic malignancies, three phase I/II clinical trials are currently recruiting patients to study the potential use of TLR3, TLR4, and TLR9 agonists in combination with immunotherapeutics and radiation therapy for the treatment of recurrent low-grade B-cell lymphomas, T-cell lymphoma, and follicular non-Hodgkin’s lymphoma (Clinical Trial Identifiers: NCT02254772, NCT02501473, and NCT01976585) (102–104).

## Summary and Future Directions

**Figure 2:**
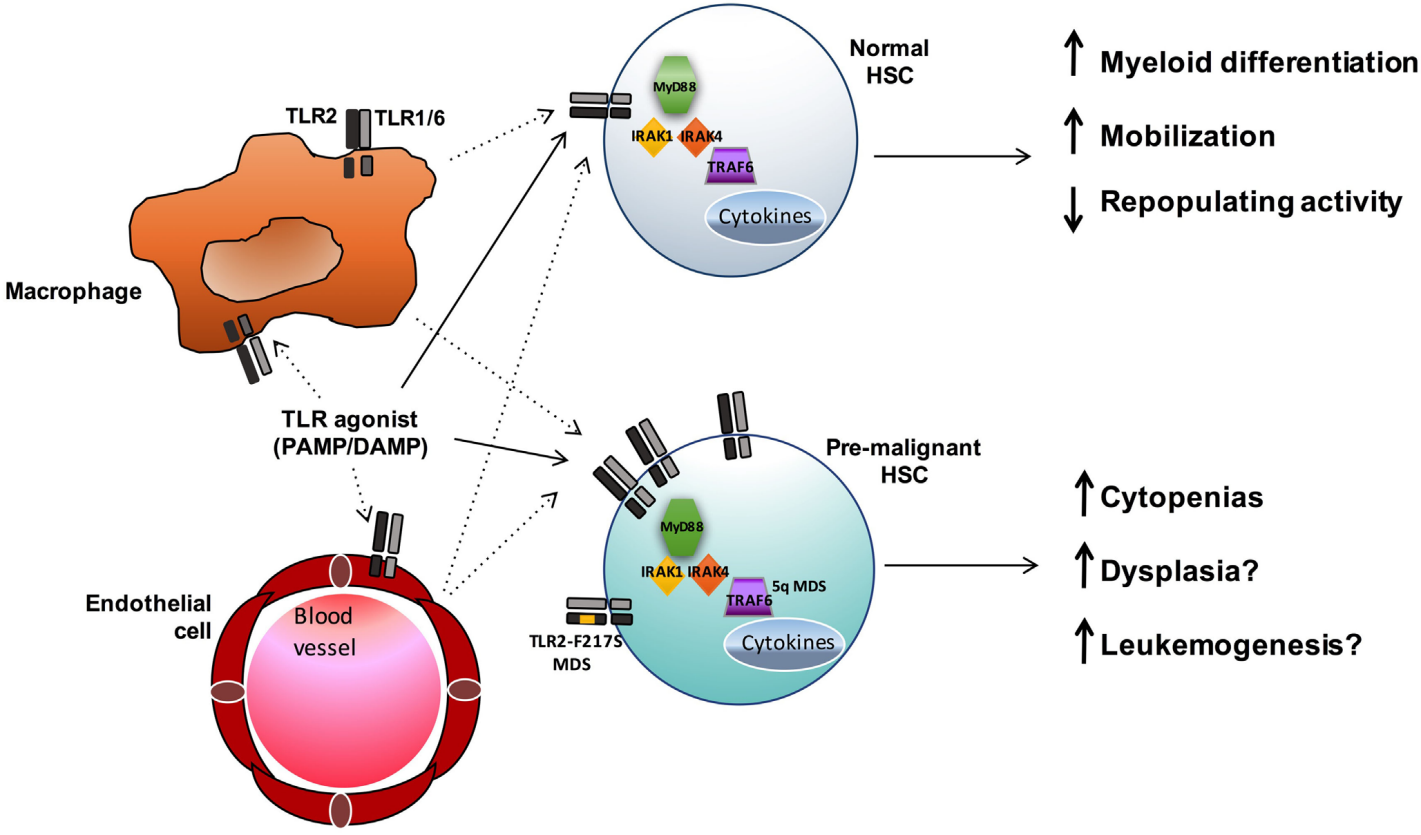
**TLR signaling influences both normal and premalignant HSCs**. TLR agonists may both directly (solid lines) and indirectly (dashed lines) influence HSCs. In normal HSCs, TLR signals promote myeloid differentiation and mobilization, and sustained exposure to TLR agonists can result in reduced HSC function. In MDS and other hematopoietic malignancies, enhanced TLR signaling (*via* increased expression or, in the case of MDS, acquisition of the activating TLR2-F217S variant or increased TRAF6 activity in 5q- cases) may contribute to ineffective hematopoiesis and leukemogenesis.

Toll-like receptors have a well-established role in innate immunity and inflammation upon stimulation by foreign pathogens and endogenous DAMPS. The type of ligand, in part, dictates both the subcellular localization of the TLR and the unique downstream signaling pathways that are activated, leading to numerous cellular effects, including cytokine secretion and regulation of gene expression. While the role of TLR signaling in committed effector immune cells has been extensively studied, recent evidence suggests these receptors can also influence earlier precursor HSC and progenitor populations. The expression of multiple TLRs has been described in normal HSCs as well as in stromal cells of the bone marrow niche. Upon stimulation, TLRs either directly activate HSCs or act indirectly on them through the production of proinflammatory cytokines by bone marrow niche cells or immune effector cells (Figure 2). While some degree of TLR signaling may be essential for the development of a normal immune response, accumulating data show an association between enhanced TLR expression and signaling and hematopoietic disorders and malignancies, such as MDS and lymphoid neoplasms (Table 1).

As discussed herein, numerous studies have linked altered TLR signaling to hematopoietic dysfunction and malignancy, supporting a role for this signaling in the pathogenesis of these diseases. However, in many cases, questions remain regarding the etiology of the increased TLR expression and signaling and the precise contribution of this signaling to the initiation and/or progression of disease. Inflammatory signals themselves, including cytokines such as IFN-gamma and G-CSF and TLR ligands such as LPS, have been shown to upregulate TLR expression on various bone marrow cell types (42, 105–108). Thus, a proinflammatory tumor microenvironment could contribute to the increased TLR expression seen in multiple hematopoietic malignancies. Furthermore, for some TLRs, including TLR2, 4, and 5, membrane receptor expression shifts to cytoplasmic during dysplastic transformation in solid tumors (109). In addition, endogenous ligands, including HMGB1, are capable of translocating to the cytoplasm upon malignant transformation (109, 110), suggesting the possibility that alterations in the subcellular locations of TLRs and their ligands could contribute to enhanced TLR signaling in hematopoietic malignancies. Further studies will be needed to investigate these ideas and determine the factors that influence TLR expression and signaling in premalignant and malignant hematopoietic cells.

In addition to questions regarding the stimulus for enhanced TLR expression and signaling in hematopoietic neoplasms, in many cases, the significance of this signaling to the pathogenesis of disease is not well understood, and additional studies are needed to determine the effects of augmenting or inhibiting TLR signaling on disease outcomes (i.e., severity of cytopenias, and initiation and/or progression of leukemia). Furthermore, while multiple different TLRs have been shown to have elevated expression in various tumors, it is not clear to what extent these different receptors may confer unique signals leading to the promotion of tumor development (In other words, does all TLR signaling generally have the same impact on premalignant and malignant cells, or do different TLRs ultimately influence downstream mediators that are unique to particular receptors?). Furthermore, the downstream mediators of TLR signaling that regulate HSC cycling, differentiation, and function, and that ultimately therefore may promote leukemogenesis, are not well known. And finally, the contribution of cell-autonomous versus cell non-autonomous TLR signaling to hematopoietic malignancies is not well understood. Although the expression and signaling of TLRs is known to be increased on the CD34+ cells of MDS patients, for example, as well as the leukemia cells in various myeloid and lymphoid cancers, it is not known whether other cell types within the bone marrow microenvironment (hematopoietic and stromal) also display aberrant TLR signaling, and what role this non-autonomous signaling may play in the pathogenesis of the disease.

In line with the lack of detailed understanding of the role of TLR signaling in many hematopoietic malignancies, several questions remain regarding the utility of targeting these receptors therapeutically. As discussed above, TLR expression and signaling is generally enhanced in hematopoietic neoplasms, and therefore inhibition of TLR signaling is a logical therapeutic strategy. While TLR antagonists are under investigation currently in select cases (OPN-305 in MDS and Ibrutinib in WM, for example), there are multiple studies conversely showing an antitumor effect of TLR stimulation, with TLR agonists either directly affecting cell growth and chemosensitivity or indirectly supporting tumor cell death *via* promotion of an antitumor immune response. A more defined understanding of the mechanism through which TLRs influence hematopoietic neoplasms is therefore necessary to better delineate the utility of TLR agonists versus antagonists as novel therapeutic options and balance the potential benefits of reducing hyperactivated TLR signaling on hematopoiesis with the risks of suppressing the immune response against the tumor cells as well as infections (111).

Based on the studies discussed within, it has become increasingly apparent that the effects of TLR signaling on tumor growth and progression requires careful consideration of numerous factors, including the TLR in question, unique downstream effectors, the influence of the tumor microenvironment, and the tumor type. Future studies aimed at gaining a comprehensive mechanistic understanding of the role of TLR signaling in lymphoproliferative disorders, MDS, and other hematopoietic neoplasms are required to more fully appreciate the influence of this signaling on disease pathogenesis and determine the appropriate utility of TLR agonists and antagonists as therapeutic agents.

## Author Contributions

DM, SB, and LS wrote and edited the manuscript.

## Conflict of Interest Statement

The authors declare that the research was conducted in the absence of any commercial or financial relationships that could be construed as a potential conflict of interest.
